# Role of Neurectomy in Inguinodynia Following Hernioplasty: A Randomized Controlled Trial

**DOI:** 10.7759/cureus.20306

**Published:** 2021-12-09

**Authors:** Bipin Kishore Bara, Sujit Kumar Mohanty, Satya Narayan Behera, Ashok Kumar Sahoo, Swagat Agasti, Satej Patnaik, Santanu Kumar Swain

**Affiliations:** 1 Surgery, Bhima Bhoi Medical College, Bolangir, IND; 2 Surgery, Srirama Chandra Bhanja (SCB) Medical College and Hospital, Cuttack, IND; 3 Surgery, Jawaharlal Institute of Postgraduate Medical Education & Research, Puducherry, IND

**Keywords:** visual analog scale, prophylactic, chronic groin pain, ilioinguinal nerve, lichtenstein

## Abstract

Introduction

To date, Lichtenstein tension-free mesh hernioplasty is being adopted widely for inguinal hernia repair in adults, although it is accompanied by procedural complications such as recurrences, infection, testicular atrophy, post-operative pain, and nerve injury. As the recurrence rate decreased after Lichtenstein's tension-free hernioplasty, surgeons’ point of focus shifted more toward postoperative groin pain (inguinodynia) after inguinal hernia repair, as it has become a quite significant problem. The nerves of interest in the inguinal region are ilioinguinal, iliohypogastric, genitofemoral, and lateral femoral cutaneous nerves. Out of all the nerves, the ilioinguinal nerve is at the greatest risk of entrapment during meshplasty. Chronic groin pain is quite significant following hernia repair, and irrespective of the severity, it can interfere with normal daily activity. The traditional surgical technique recommends the preservation of the ilioinguinal nerve to avoid the morbidity associated with the cutaneous sensory loss supplied by the nerve. One popular belief is that if we excise the ilioinguinal nerve, then the chance of getting post-operative neuralgia due to entrapment, inflammation, neuroma, or fibrotic reactions will almost become zero. Hence, this study was conducted to evaluate the effect of prophylactic excision of the ilioinguinal nerve in the patients undergoing Lichtenstein hernioplasty for inguinal hernias.

Methods

All consecutive male patients presenting to the Department of Surgery with inguinal hernia and age above 18 years were included in the study. All the patients were operated on under spinal anesthesia. Lichtenstein tension-free hernia repair was taken as the standard procedure for hernia repair. Patients in whom the nerve was preserved were kept in group A, whereas group B comprised patients who had undergone neurectomy. Patients were followed up regarding pain at first, third, and sixth months, at rest, and after exercise. The pain was graded according to the VAS (visual analog scale) scoring.

Results

In the present study, out of a total of 92 patients, 80 patients were included. In the first month, 15% of the patients in group A had mild pain, while 5% in group B had experienced a moderate degree of pain at rest. After exercise, the result was 30% in group B. Similarly, in the third month of follow-up, it was found that 25% of the patients in group A experienced mild pain, while 12.5% complained about a moderate degree of pain who had to take analgesics for a longer period. After putting them to exercise and then grading the pain, it was found that 32.5% in group A and 15% in group B experienced pain. After follow-up for six months in both groups, it was revealed that there was no significant difference in post-operative pain at rest (10% and 7.5% in groups A and B, respectively). After exercise, 20% of patients in group A complained of pain, while in group B, only 10% experienced pain. There was no significant difference between both the groups while comparing chronic groin pain at rest and after exercise, and after different time intervals in follow-up (p = 0.4513 and 0.548, respectively).

Conclusion

Prophylactic excision of the ilioinguinal nerve in Lichtenstein tension-free meshplasty decreased the incidence of chronic groin pain after surgery but it was statistically insignificant. Furthermore, this procedure did not affect the quality of life after surgery.

## Introduction

By definition, a protrusion of a viscous or a part of viscous through an abnormal opening in the walls of its containing cavity is called a hernia [1]. There are many classifications for hernias, but the Nyhus classification is accepted worldwide [2]. Inguinal hernia is the most common abdominal wall hernia and accounts for 80% of the total hernias in adults [3]. Around 90% of all groin hernias are inguinal hernias, which affect 25% of adult males in their lifetime [4]. Men are 25 times more likely to have a groin hernia than women. An indirect inguinal hernia is the most common hernia, regardless of gender. In men, indirect hernias predominate over direct hernias at a ratio of 2:1 [5]. History has witnessed many interesting facts about hernia treatment, starting from medical conservative therapy to the evolution of different surgical techniques. It was Bassini who proposed a comprehensive understanding of inguinal anatomy and a successful hernia repair with minimal morbidity. Tissue-based repair of McVay, Shouldice, and even modern Desarda techniques are considered derivatives of Bassini. But the revolutionary change in the treatment of hernia occurred after Lichtenstein first introduced meshplasty for the tension-free repair of hernia. To date, Lichtenstein tension-free mesh hernioplasty is being adopted widely for inguinal hernia repair in adults, although it is accompanied by procedural complications such as recurrences, infection, testicular atrophy, post-operative pain, and nerve injury. As the recurrence rate decreased after Lichtenstein's tension-free hernioplasty, surgeons’ point of focus shifted more toward post-operative groin pain (inguinodynia) after inguinal hernia repair, as it has become a quite significant problem, i.e., 63% of the patients experience postoperative groin pain [6].

Chronic groin pain following inguinal hernia repair is defined as pain lasting more than six months after surgery [7]. Chronic groin pain can be classified into neuropathic, nociceptive (somatic), and visceral pain [8].

Nociceptive pain is the most common of the three, and the etiology is fibrosis over the mesh (more in the small pores compared to the large pores), mechanical pressure by folding of the mesh by displacement or contraction of the mesh. Nerves may get injured by surrounding structures such as periosteal layers or musculotendinous tissue or due to post-operative causes [9]. Visceral pain is felt due to the sympathetic plexus injury and may be felt during ejaculation as the pain travels by the afferent autonomic fibers.

Neuropathic pain occurs due to direct nerve injuries by either accidental cutting or excessive traction. It may also happen through damage by electrocoagulation, or entrapment by the perineural fibrosis, suture material, stapler, or by the mesh itself [10]. Direct nerve injury causes partial or complete resection of nerves.

The nerves of interest in the inguinal region are ilioinguinal, iliohypogastric, genitofemoral, and lateral femoral cutaneous nerves. Ilioinguinal and iliohypogastric nerves are encountered in the anterior dissection, and the last two nerves become endangered during laparoscopic repair of the inguinal hernia. Despite the anatomic variation among the nerves in the inguinal region, all the nerves are identifiable individually in 70% to 90% of cases. Out of all the nerves, the ilioinguinal nerve is at the greatest risk of entrapment during meshplasty.

The ilioinguinal nerve (L1) arises from the anterior ramus of the first lumbar nerve root in the lumbar plexus. After exiting the plexus, it then travels across the quadrates lumborum as it courses laterally and passes the iliacus as it approaches the iliac crest. Then it wraps anteriorly and pierce the transverse abdominis and internal oblique muscle (posterior wall of the inguinal canal) to become the content of the canal. It travels in the inguinal canal with the spermatic cord until it leaves a superficial inguinal ring. It provides a sensory supply to the skin of the upper and medial parts of the thigh. In males, it supplies the root of the penis and the upper part of the scrotum, while in females it innervates the mons pubis and labium majus.

As per the report, chronic groin pain is quite significant, ranging from 19% to 62.9% following hernia repair, and irrespective of the severity, it can interfere with normal daily activity [11]. Hence, it is a challenge for the surgeons to treat this problem, which is often very difficult.

There are two types of options to treat the long-term complications of post-operative inguinodynia, i.e, non-surgical and surgical. Non-surgical options include the use of analgesics (NSAIDS, antidepressants), a peripheral nerve block with local anesthesia, transcutaneous electric nerve stimulation, laser therapy, pulsed radiofrequency, whereas surgical options include revise operation for recurrence and meshoma, removal of fixation material, selective neurolysis or neurectomy of ilioinguinal, iliohypogastric, or genitofemoral nerve, and removal of mesh and further hernia repair.

The traditional surgical technique recommends preservation of the ilioinguinal nerve to avoid the morbidity associated with the cutaneous sensory loss supplied by the nerve to the groin, medial aspect of the thigh, upper part of the scrotum, and the root of the penis.

One popular belief is that if we excise the ilioinguinal nerve, then the chance of getting post-operative neuralgia due to entrapment, inflammation, neuroma, or fibrotic reactions will almost become zero [12]. Hence, this study was conducted to evaluate the effect of prophylactic excision of the ilioinguinal nerve in the patients undergoing Lichtenstein hernioplasty for inguinal hernias.

## Materials and methods

The study was conducted in the Department of Surgery at a tertiary care hospital in Eastern India from February 2019 to January 2020. The study was approved by the Ethics Committee of the institute (Bhima Bhoi Medical College & Hospital, Balangir, Odisha, with project no-9/05.02.2019) and has been performed following the ethical standards laid down in an appropriate version of the Declaration of Helsinki (as revised in Brazil 2013).

All consecutive male patients presenting to the Department of Surgery with inguinal hernia and age above 18 years were included in the study. The details of the patients and the findings were recorded. Patients with diabetes mellitus, complicated inguinal hernia (obstructed/strangulated), bilateral inguinal hernia, previous surgeries with lower abdominal or paramedian incision or incision in the inguinal region, recurrent inguinal hernia, mesh allergy and subsequent hernia repair, previous history of trauma and pain in the inguinal region, history of low back pain, and any previous spinal disorders were excluded from the study.

The study was designed as a prospective, open-labeled, parallel-arm, randomized controlled trial. Block randomization was carried out using a computer program with randomly selected block sizes of four and six. Allocation concealment was ensured by a serially numbered opaque-sealed envelope (SNOSE).

All consecutive patients with inguinal hernia were recruited in the primary cohort after obtaining written informed consent.

Patients were selected after thorough history taking, clinical examination, laboratory investigations, and radiological investigation (chest X-ray and sonography of the abdomen & pelvis). All the patients were operated on under spinal anesthesia. Lichtenstein tension-free hernia repair was taken as the standard procedure for hernia repair. Patients in whom the nerve was preserved were kept in group A, and group B comprised patients who had undergone neurectomy.

Careful dissection of the inguinal canal was performed layer by layer. The ilioinguinal nerve was identified and preserved in group A patients, whereas, in group B, it was cut near the deep inguinal ring. The nerve was identified and stretched back to the point where it came out of the internal oblique near the deep ring. Then it was transected and ligated, and the proximal ligated end was buried in the internal oblique muscle to prevent neuroma formation. At the medial end, the nerve was cut very close to the lateral border of the rectus muscle (the desired effect was obtained by pulling the nerve laterally). Lichtenstein tension-free mesh repair was performed using a polypropylene mesh of size 7x15 cm, which was again cut into the appropriate size of the posterior wall and was placed in position using Prolene 2-0 (Ethicon Inc., Somerville, NJ) non-absorbable suture.

Patients were given antibiotics and analgesics for five days to decrease post-operative pain and infection. Suture were removed between 8 and 10 days depending on the condition of the surgical wound. Follow-up was done regarding pain at first, third, and sixth months, at rest, and after exercise (coughing 10 times, walking up three flights of the staircase, and cycling for 10 minutes). The pain was graded according to the VAS (visual analog scale) scoring [13].

Statistical analysis

Data were analyzed using SPSS Statistics for Windows, Version 20.0 (IBM Corp., Armonk, NY). Appropriate statistical tests were used to compare the results of groups A and B. A p-value of <0.05 was considered statistically significant.

## Results

**Figure 1:**
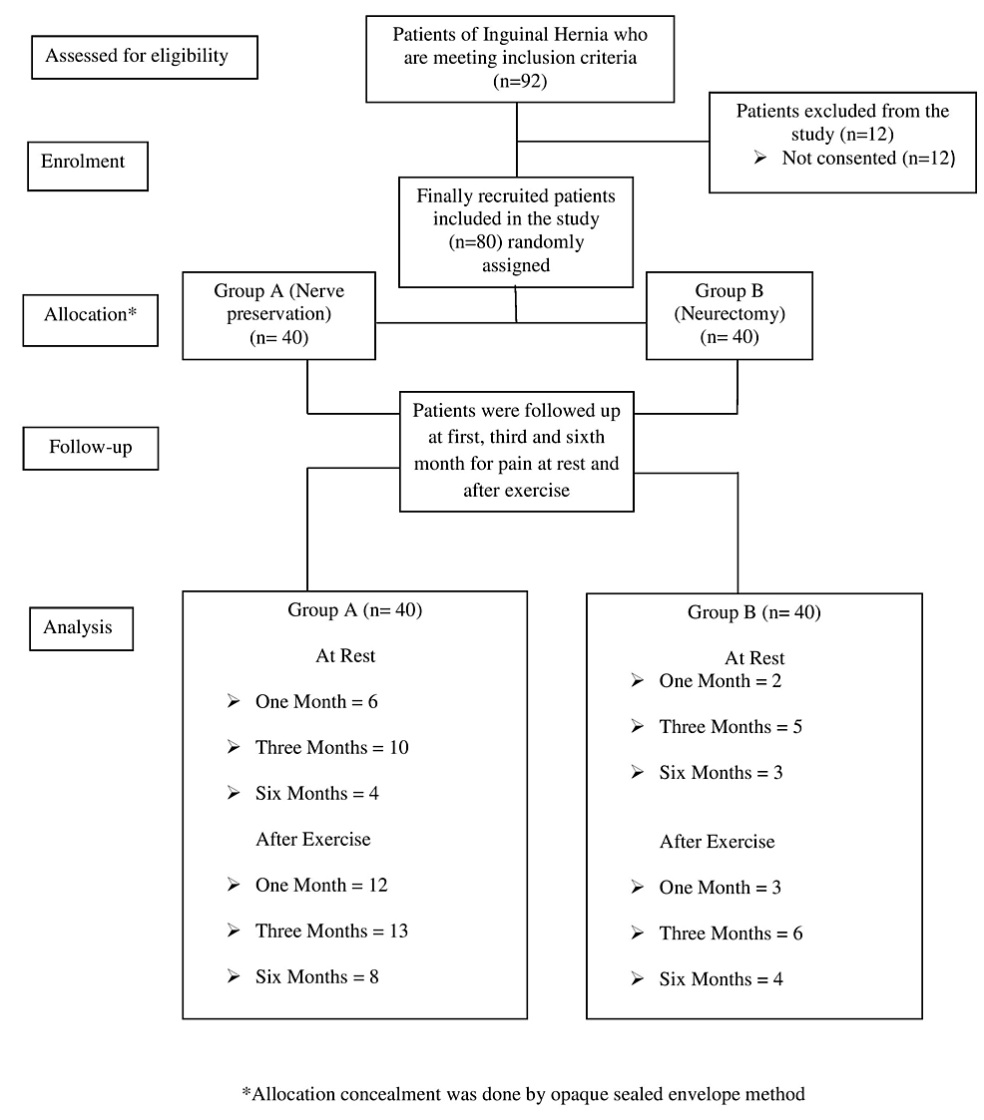
The overall scheme as per the CONSORT flowchart CONSORT, CONsolidated Standards Of Reporting Trials

In the present study, out of a total of 92 patients, 80 patients were included (Figure 1).

Due to non-agreement to participate in the study, 12 patients were excluded. The demographic profile of the patients in both groups was comparable. On immediate post-operative days, there was no significant difference in pain between both the groups till the removal of stitches and discharge of the patients. The chronic groin pain at rest and after exercise are given in Tables 1, 2.

**Table 1:** Incidence of chronic groin pain at rest

Time of study	Group A	Group B	p-Value
One month	15% (6)	5% (2)	0.4513
Three months	25% (10)	12.5% (5)	
Six months	10% (4)	7.5% (3)	

**Table 2:** Incidence of chronic groin pain after exercise

Time of study	Group A	Group B	p-Value
One month	30% (12)	7.5% (3)	0.548
Three months	32.5% (13)	15% (6)	
Six months	20% (8)	10% (4)	

However, at first month, 15% of the patients in group A had mild pain, while 5% in group B had experienced a moderate degree of pain at rest. After exercise, the result was 30% in group B.

Similarly, in the third month of follow-up, it was found that 25% of the patient in group A experienced mild pain, while 12.5% complained about a moderate degree of pain and had to take analgesics for a longer period. After putting them to exercise and then grading the pain, it was found that 32.5% in group A and 15% in group B experienced pain.

After follow-up for six months in both groups, it was revealed that there was no significant difference in post-operative pain at rest; it was 10% and 7.5% in groups A and B, respectively. But when they were subjected to minimal exercise, there was a significant difference in results. 20% of patients in group A complained of pain, while in group B only 10% experienced pain.

There was no significant difference between both the groups while comparing chronic groin pain at rest and after exercise after different time intervals in follow-up (p = 0.4513 and 0.548, respectively).

In the present study, there were neither major complications nor any complaints regarding the recurrence of the hernia. Ilioinguinal nerve excision was not associated with any reduction in the quality of life assessed during the follow-up.

## Discussion

As recurrence rates of inguinal hernia have dramatically reduced after the introduction of meshplasty, post-operative chronic groin pain becomes the next concern for the surgeons as it is one of the most common complaints that affects the patient's satisfaction and quality of life after hernioplasty [10,14]. The mechanism for post-operative groin pain is due to inflammation and fibrosis caused by the mesh that is near the ilioinguinal nerve [15].

Ravichandran et al. were the first to study the effect of ilioinguinal neurectomy [16]. They performed the trial in the patients with a bilateral inguinal hernia in which they performed neurectomy on one side and preservation of the nerve on another side. The trial was found out to be a non-inferiority trial as there was no such evidence to support the benefit of ilioinguinal neurectomy. This study was limited by its small sample size and short follow-up period. Also, they did not compare between two groups and did not study the chronic groin pain after various exercises.

In another study conducted by Dittrick et al. who randomly divided 156 patients into two groups by performing nerve preservation and nerve transection, there was no significant difference between both the groups after one month of follow-up [17]. They also did not study groin pain, numbness, or pain after various exercises.

In the present study, the post-operative pain at rest after one month was 5% in group B population, while it was more (15%) in group A. The pain became exaggerated in both groups after exercise (30% vs. 7.5% in groups A and B, respectively).

Similarly, at three months post-operation, groin pain at rest was found in 12.5% of the group B population as compared to 25% of the group A population. This change became more significant when both groups were studied after exercise. The results obtained were 15% in group B vs. 32.5% in group A; these results are comparable with those in the study conducted by Malekpour et al. in 2008 (12.5% [nerve preservation] vs. 26.9% [nerve division]) [18].

At six months, this study showed a result of 7.5% in group B vs. 10% in group A at rest and after exercise, the result became 10% in group B vs. 20% in group A, which was nearly the same as the results obtained by Mui et al. in 2006 (19.2% [nerve preservation] vs. 8.2% [nerve division]) [19].

There was no significant difference between both the groups while comparing chronic groin pain at rest and after exercise after different time intervals in follow-up (p = 0.4513 and 0.548, respectively).

In this study, we did not consider the pain experienced during the first week as it might be due to an operative procedure or infection. It was controlled by the use of antibiotics along with analgesics till the removal of stitches.

There are a few limitations in the present study. The sample size of the study was small and it was a single-center study.

## Conclusions

The results of the study revealed that prophylactic excision of the ilioinguinal nerve in Lichtenstein tension-free meshplasty decreased the incidence of chronic groin pain after surgery, but it was statistically insignificant. Furthermore, this procedure did not affect the quality of life after surgery. This procedure also had no specific complications. As the sample size was less, further studies may be needed to establish the conclusion of the study.
